# CBCT Evaluation of the maxillary palatine process as a donor site for the regeneration of periodontal defects

**DOI:** 10.34172/japid.2022.006

**Published:** 2022-04-20

**Authors:** Yaser Safi, Sepideh Behzadi, Marziyeh Shafizadeh, Reza Amid, Mahdi Kadkhodazadeh

**Affiliations:** ^1^Department of Radiology, School of Dentistry, Shahid Beheshti University of Medical Sciences, Tehran, Iran; ^2^Department of Restorative Dentistry, School of Dentistry, Shahed University of Medical Sciences, Tehran, Iran; ^3^Dental Research Center, Shahid Beheshti University of Medical Sciences, Tehran, Iran; ^4^Department of Periodontics, School of Dentistry, Shahid Beheshti University of Medical Sciences, Tehran, Iran

**Keywords:** Autografts, bone transplantation, cone-beam computed tomography, maxilla, palate

## Abstract

**Background:**

The maxillary palatine process (MPP) is an excellent source of autogenous bone transplants for anterior maxillary reconstruction. This research aimed to determine the quantity and quality of accessible MPP as a donor location.

**Methods:**

Cone-beam computed tomography (CBCT) scans of patients referred to the School of Dentistry were evaluated by a certified examiner. The harvestable MPP was defined as the space between the distal surfaces of maxillary first premolars. OnDemand 3D Imaging software was used to determine bone properties. SPSS software was used to investigate the following variables: Bone characteristics are correlated with age and gender, accessible volume, and palatal width and height. *P*<0.05 was defined as the level of statistical significance.

**Results:**

This study was performed on CBCT scans of 81 subjects (41 females and 40 males). MPP volume and palatal heights were 1.33±0.53 cm3 and 20.86±5.51 mm, respectively. Maximum bone density was observed around lateral incisors. Palatal width was 33.81±2.42 mm between canines and 41.81±2.66 mm between premolars. The MPP volume was significantly greater in males (*P*<0.001). Additionally, there was a positive correlation between the volume and palatal width (*P*<0.05).

**Conclusion:**

Within the constraints of this research, there is a limited supply of MMP accessible for use as a graft source, and it is best suited for treating localized bone lesions. The favorable link between palatal breadth and harvestable volume aids the surgeon in estimating the quantity of bone accessible during the first evaluation.

## Introduction

 Significant recession in the alveolar bone often develops as a result of tooth extraction and sinus pneumatization.^1-3^ Implant insertion in a damaged ridge may jeopardize the aesthetic outcome and reduce the success of the operation.^4^ As a result, before implant placement, bone regeneration of an overly resorbed alveolar ridge may be essential.

 Numerous graft sources have been proposed for guided tissue regeneration, including alloplastic, allogenic, and autogenous bone grafts.^5,6^ Each source has advantages and limitations; nonetheless, owing to its high success rate, autogenous bone graft remains the gold standard for alveolar bone regeneration.^7^ There are two kinds of autogenous donor sites: intraoral and extraoral. Intraoral sites are preferred for oral rehabilitation because they are close to the recipient site, provide comfort for the patient, and do not need hospitalization.^8^ Several donor sites may be employed depending on the size and location of the defect, including the coronoid process, mandibular ramus and symphysis, maxillary tuberosity, and maxillary palatine process (MPP).^9,10^ Despite various associated difficulties such as sensory abnormalities, the mandibular ramus and symphysis are often employed for grafting localized oral lesions.^11-13^ Using the MPP as a donation site has several benefits. The most significant advantage occurs when the recipient site is located in the anterior maxilla since this minimizes the operational time and morbidity associated with a second flap in the donor location.^14^

 The amount and quality of bone available for clinical use as a donor site are critical criteria for making an informed clinical decision.^15,16^ Additionally, bone quality affects the expected outcome of grafting.^17^ Given the critical role of bone properties in the grafting procedure and the limited evidence about the MPP, this research used cone-beam computed tomography (CBCT) scans to determine the amount and quality of harvestable palatine bone. Additionally, the purpose was to determine the relationship between bone properties and gender and age.

## Methods

 The Ethics Committee of the Dental School at Shahid Beheshti University of Medical Sciences in Tehran, Iran, authorized this cross-sectional research (3IR.SBMU.DRC.REC.1398.01). The protocol for the research followed the Declaration of Helsinki. Between 2016 and 2017, patients were sent to Shahid Beheshti University’s School of Dentistry. The CBCT scans were taken for various dental procedures (orthodontic treatment and implant placement), not for this research. In addition, all the patients signed informed consent forms to participate in this investigation.

 The following criteria for exclusion were used: systemic conditions affecting the bone structure, such as osteoporosis, thalassemia, and sickle cell disease, rheumatoid arthritis, scleroderma, any signs of trauma, cysts, tumors, or pathologic defects detected on CBCT, cleft palate, severe periodontitis, impacted tooth in the area, or a history of orthodontic treatment or orthognathic surgery. Finally, 81 adult individuals’ radiography records were included. The CBCT scans were acquired using a single CBCT machine (NewTom VG, QR srl/AFP Imaging Co.) operating at 90 kV, 8 mA, and face mode with a field of view (FOV) of 7.510 cm and a voxel size of 0.2 mm. To perform measurements, the main scans were converted to the Digital Imaging and Communications in Medicine (DICOM) format and opened in Cybermed’s OnDemand 3D software (Seoul, Korea).

**Figure 1:**
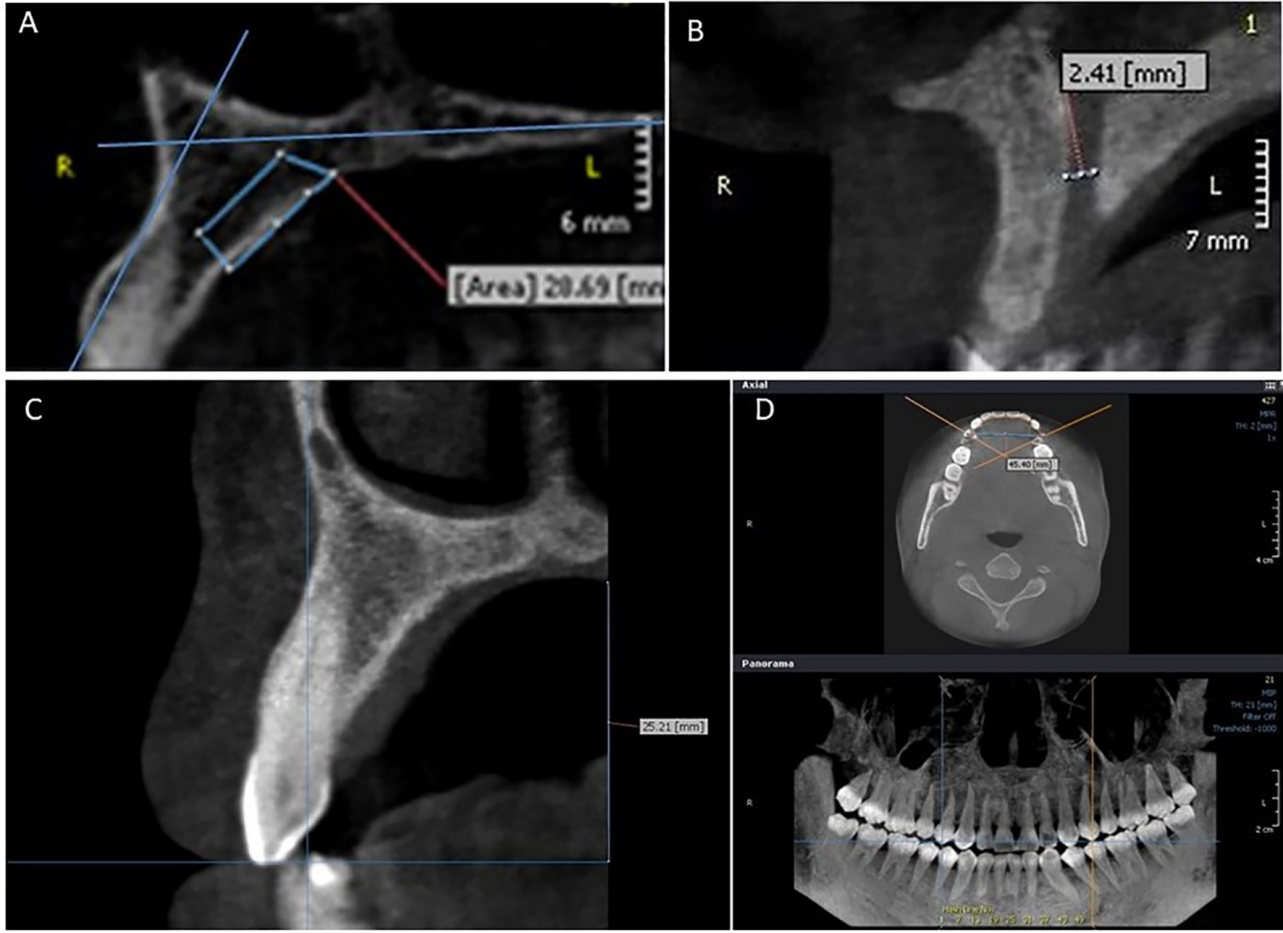
A. Sagittal cross-section of the harvestable maxillary palatine process. B. Incisive canal diameter. C. Palatal height. D. Palatal width at canine and premolar sites. The intersection point of the crossing lines with the first premolars indicates the exact position of the buccal cusp.

 A panoramic curve was reconstructed in the axial view for obtaining sections. By default, each slice had a 1-mm thickness and was parallel to the respective tooth axis. The slices were collected from the distal aspect of the first premolar on one side to the distal aspect of the second premolar on the other side. Every irrelevant region was omitted in the sagittal view. The harvestable area was identified using an earlier work by Hassani et al.^18^Figure 1A illustrates the surface of the harvestable MPP in a sagittal view. A line was drawn parallel to the tooth axis, and a line was drawn at 2 mm from the nasal cavity floor. The harvestable area was defined by the following lines: The upper margin was at 2 mm from the nasal cavity floor and maxillary sinus, the frontal margin was at 2 mm from the tooth surface, and the posterior margin was defined by drawing a line from the upper margin (at the most posterior point) perpendicular to the hard palate. To establish a 2-mm safety margin from the incisive canal, the last slice in which the incisive canal was observed was found, and the two following slices were removed. The surface of the target area was measured in every slice. Then, the volume between every two slices was measured using the partial frustum formula:^19^


$$v=\frac{h}{3}\left(s1+s2+\sqrt{s1+s2}\right)$$

 In this formula, h refers to the thickness of each interval (1 mm), and s1 and s2 indicate surfaces measured in two consecutive slices. Then, the following parameters were determined:

 1. The diameter of the incisive canal was measured perpendicular to the canal’s buccal and palatal cortical plates (Figure 1B). This measurement was taken at the canal’s widest point.

 2. The bone density was determined around lateral incisors, canines, and first premolars. The measurements were performed using the ROI tool in the OnDemand software and the gray value (GV) scale.

 3. Palatal height was measured from the soft palate to the canine cusp (Figure 1B). This measurement was performed in a slice where the canine was observed at the greatest length. A line was drawn from the canine cusp parallel to the palate, and the distance between this line and the palate was measured.

 4. Palatal width was determined between canines and premolars in the axial view (Figure 1C). Measurements were performed from the buccal cusp of the first premolars and canines. To increase the precision of measurements, in the panoramic view, cross lines were drawn at the middle of canines and first premolars to determine the exact position of cusps in the axial view.

 A qualified examiner (Behzadi, S.) performed all the measurements. To confirm the reliability, the values of 10 samples were measured repeatedly two weeks after the initial evaluation. An intraclass correlation coefficient (ICC) of >0.8 was obtained, indicating acceptable intraexaminer reliability.

 Data analyses were performed to evaluate the association of bone characteristics with age and gender and the correlation between volume and palatal width. Means and standard deviations (SD) were calculated for every parameter. The Kolmogorov-Smirnov test was used to investigate data normality. Normal variables were then analyzed using Pearson’s correlation coefficient and independent t-test. Finally, the Spearman and Mann-Whitney correlation tests were used for abnormal variables. All the tests were carried out by SPSS 19, and P<0.05 was considered statistically significant.

## Results

 Eighty-one individuals were included in this study (41 females and 40 males). The patients presented tooth integrity in the area of interest (teeth #14 to #24), with a median age of 38.12±12.51 years (range: 20‒70). The mean and standard deviation values for the bone parameters tested are presented in Table 1. The mean volume of harvestable bone in this group was 1.33 [0.53] cm^3^. Palatal width between canines was 33.81 [2.42] mm, Palatal width between premolars was 41.81 [2.66] mm, and palatal height was 20.86 [5.51] mm. The greatest density was observed at the site of lateral incisors (622.17 [321.41] GV).

**Table 1:** Mean, standard deviation (SD), minimum, and maximum values of parameters

**Variable**	**Mean**	**SD**	**Min**	**Max**
**Incisive canal width (mm)**	2.35	0.96	0.68	4.52
**Volume (cm** ^3^ **)**	1.33	0.53	0.32	3.26
**Density in the lateral incisor area (GV)**	622.17	321.41	11.30	1740.00
**Density in the canine area (GV)**	602.37	317.85	25.00	1672.00
**Density in the first premolar area (GV)**	498.89	291.35	10.00	1467.00
**Palatal height (mm)**	20.86	5.51	9.85	38.94
**Palatal width in the canine area (mm)**	33.81	2.42	27.15	40.39
**Palatal width in the first premolar area (mm)**	41.81	2.66	32.38	49.61

GV: Gray value

Table 2 illustrates the relationship between bone properties and gender. Gender had a significant relationship with available bone volume (P<0.001); the mean volume in men was 0.43 cm^3^ on average, more than that in females. In addition, males had substantially wider palatal widths in canine and first premolar areas (1.18 mm and 1.49 mm, respectively; P<0.05).

**Table 2:** Correlation of bone parameters with gender

**Variable**	**P-value**
**Incisive canal width (mm)**	0.137^1^
**Volume (cm** ^3^ **)**	P<0.001^1*^
**Density in the lateral incisor area (GV)**	0.555
**Density in the canine area (GV)**	0.647^1^
**Density in the first premolar area (GV)**	0.887^1^
**Palatal height (mm)**	0.947^1^
**Palatal width in the canine area (mm)**	0.027^*^
**Palatal width in the first premolar area (mm)**	0.012^*^

1Calculated using the Mann-Whitney test; the remaining values were calculated using the t-test. *significant relationship (P<0.05). GV: Gray value

 There was no significant correlation between age and bone parameters (Table 3).

**Table 3:** Correlation of bone parameters with age

**Variable**	**P-value**	**Correlation coefficient**
**Incisive canal width (mm)**	0.112	0.178
**Volume (cm** ^3^ **)**	0.933	-0.009
**Density in the lateral incisor area (GV)**	0.308^1^	0.115
**Density in the canine area (GV)**	0.385	0.098
**Density in the first premolar area (GV)**	0.344	0.107
**Palatal height (mm)**	0.546	-0.068
**Palatal width in the canine area (mm)**	0.211^1^	0.14
**Palatal width in the first premolar area (mm)**	0.25^1^	0.129

1Calculated using the Pearson’s correlation test; the remaining values were calculated using the Spearman test. No significant relationship was found (P<0.05). GV: Gray value

 Spearman’s correlation coefficient demonstrated a statistically significant association between bone volume and palatal width in both canine and first premolar areas (P<0.05). There was no statistically significant association between available bone volume and palatal height (P=0.45).

## Discussion

 There are various benefits to adopting intraoral donor sites for grafting, including easier surgical access, no danger of cutaneous scarring, and outpatient surgery.^8^ A more accurate assessment of the bone’s quality and accessible volume may assist the surgeon in selecting a more acceptable grafting source. As a result, this research evaluated the properties of harvestable MPP.

 The mean volume of the maxillary palatine process was measured in this research at 1.33 0.53 cm^3^. Bernades-Mayordomo et al.^10^ reported a mean volume of 2.41±0.78 cm^3^ in dentate patients, measured through a reconstruction of harvestable MPP by 3-D imaging software. Hassani et al.^18^ found comparable findings in a study of dry skulls. They found a mean volume of 2.03 cm^3^ in dentate and 2.40 cm^3^ in edentulous subjects. The disparity in measurements may be due to ethnic anatomical differences, unequal sample numbers, or measuring methodologies. Verdugo et al.^20^ revealed in a study on ramus bone that harvestable quantities determined by software design may be much less than those collected intra-operatively. While the average quantity of MPP accessible is equivalent to that of mandibular ramus and symphysis,^20-22^ it might be a better option when the recipient location is in the anterior maxilla.

 The current investigation determined the diameter of the incisive canal to be 2.35±0.96 mm. Khojastepour et al^23^ used CBCT scans to analyze the nasopalatine canal and determined its diameter at 3.17±1.01 mm, consistent with the findings of Acar et al,^24^ who discovered a mean incisive foramen diameter of 3.96 mm in the presence of central incisors and 3.79 mm in the absence of central incisors. The difference between this study and the previous studies could be due to the measurement methods. In this study, the diameter was measured in a line perpendicular to the cortical plates, while the previous studies measured the opening diameter of the canal. Unlike our results, both investigations found a substantial association between diameter and gender, and age.

 According to the findings of this research, men had a considerably wider palatal width; however, there was no correlation between palatal height and gender, consistent with earlier research.^25,26^ On the other hand, this study showed no correlation between any of the bone parameters and age, in contrast to previous reports on several other bone structures.^27,28^

 There was a substantial positive relationship between palatal width and accessible bone volume, as determined by the study. As a result, the practitioner may estimate the bone volume during the first examination by checking the palatal width. The palatal width was determined at two locations (canines and premolars) to ensure that the other could be utilized if one site had a lost tooth.

 Numerous methods have been developed for assessing the volume of bodily organs. The partial frustum model is used by splitting the volume into smaller frustums, measuring the volume of each frustum, and combining the measured results.^19^ This technique was used because of its alleged high accuracy and reliability.^29,30^ Additionally, density has conventionally been measured on Hounsfield scale (HU) using a CT scan. However, measurement of the bone density in gray value (GV) using CBCT scans is comparable to the Hounsfield scale.^17^ Since CBCT scans are more commonly prescribed in dentistry and have convincing accuracy, the GV scale was applied to determine bone density in this study.

 It should be pointed out that bone harvesting from MPP is a delicate procedure with a risk of numerous morbidities. The anatomic constraints of this region include the incisive canal, tooth surface, and maxillary sinus. Multiple variables, including the buccopalatal inclination of the maxillary anterior teeth and the sagittal skeletal profile, might alter the palatal bone thickness.^31,32^ Additionally, the remaining ridge accessible for implant placement should be addressed; if bone harvesting from this location reduces the ridge and jeopardizes implant placement, another graft source should be investigated.

 There are several limitations to this study. The major limitation of this study was the lack of advanced software which could provide more precise volume measurements compared with the manual method. In addition, although using CT scans is the first choice for assessing bone density, CBCT scans were used in this study since they are preferred in dentistry due to their lower radiation dose for the patients.^33^ Finally, compared with previous studies on the dry skull, some limitations related to CBCT acquisition from patients should be considered in this study. Limitations of CBCT acquisition from patients, such as head position, could have potentially affected the precision of scans and measurements. This might have served as a cofounding factor, resulting in differences between the measures of this study and previous studies on the dry skull.

## Conclusion

 Given its modest volume, the palatine process of the maxilla is most suited for grafting localized defects in the front area (a mean volume of 1.33 cm^3^). CBCT is still highly recommended to evaluate the exact volume in each individual and the proximity to anatomic structures. A positive correlation between palatal width and available volume enables the surgeon to estimate the available amounts at the initial examination. Finally, bone volume and palatal width were greater in men, while there was no association between any of the MPP parameters with age.

## Authors’ contributions

 Y.S. and R.A. conceived the idea. S.B. and M.S. established the theory and executed the research. M.K. verified the analytical methods. All authors read and approved the final manuscript.

## Funding

 This study did not receive any financial support.

## Availability of data

 The data used to support the findings of this study are available from the corresponding author upon request.

## Ethics approval

 This cross-sectional study was approved by the Ethics Committee of the Dental School, Shahid Beheshti University of Medical Sciences, Tehran, Iran (3‏IR.SBMU.DRC.REC.1398.01). The study protocol was in accordance with the Declaration of Helsinki.

## Competing interests

 The authors declare that they have no known competing and financial interests or personal relationships that could have influenced the work reported in this paper.
